# Isolation and Characterization of Mouse Monoclonal Antibodies That Neutralize SARS-CoV-2 and Its Variants of Concern Alpha, Beta, Gamma and Delta by Binding Conformational Epitopes of Glycosylated RBD With High Potency

**DOI:** 10.3389/fimmu.2021.750386

**Published:** 2021-10-26

**Authors:** Sabrina Mariotti, Antonio Capocefalo, Maria Vincenza Chiantore, Angelo Iacobino, Raffaela Teloni, Maria Laura De Angelis, Alessandra Gallinaro, Maria Franca Pirillo, Martina Borghi, Andrea Canitano, Zuleika Michelini, Melissa Baggieri, Antonella Marchi, Paola Bucci, Paul F. McKay, Chiara Acchioni, Silvia Sandini, Marco Sgarbanti, Fabio Tosini, Antonio Di Virgilio, Giulietta Venturi, Francesco Marino, Valeria Esposito, Paola Di Bonito, Fabio Magurano, Andrea Cara, Donatella Negri, Roberto Nisini

**Affiliations:** ^1^ Dipartimento di Malattie infettive, Istituto Superiore di Sanità, Roma, Italy; ^2^ Dipartimento Sicurezza alimentare, nutrizione e sanità pubblica veterinaria, Istituto Superiore di Sanità, Roma, Italy; ^3^ Dipartimento di Oncologia e Medicina Molecolare, Istituto Superiore di Sanità, Roma, Italy; ^4^ Centro nazionale per la salute globale, Istituto Superiore di Sanità, Roma, Italy; ^5^ Department of Infectious Disease, Imperial College, London, United Kingdom; ^6^ Centro per la sperimentazione ed il benessere animale, Istituto Superiore di Sanità, Roma, Italy; ^7^ Centro nazionale per il controllo e la valutazione dei farmaci, Istituto Superiore di Sanità, Roma, Italy

**Keywords:** SARS-COV-2 variants, neutralizing monoclonal antibodies, epitopes expression, therapy, diagnosis, pathogenesis

## Abstract

Antibodies targeting Receptor Binding Domain (RBD) of SARS-CoV-2 have been suggested to account for the majority of neutralizing activity in COVID-19 convalescent sera and several neutralizing antibodies (nAbs) have been isolated, characterized and proposed as emergency therapeutics in the form of monoclonal antibodies (mAbs). However, SARS-CoV-2 variants are rapidly spreading worldwide from the sites of initial identification. The variants of concern (VOC) B.1.1.7 (Alpha), B.1.351 (Beta), P.1 (Gamma) and B.1.167.2 (Delta) showed mutations in the SARS-CoV-2 spike protein potentially able to cause escape from nAb responses with a consequent reduction of efficacy of vaccines and mAbs-based therapy. We produced the recombinant RBD (rRBD) of SARS-CoV-2 spike glycoprotein from the Wuhan-Hu 1 reference sequence in a mammalian system, for mice immunization to isolate new mAbs with neutralizing activity. Here we describe four mAbs that were able to bind the rRBD in Enzyme-Linked Immunosorbent Assay and the transmembrane full-length spike protein expressed in HEK293T cells by flow cytometry assay. Moreover, the mAbs recognized the RBD in supernatants of SARS-CoV-2 infected VERO E6 cells by Western Blot under non-reducing condition or in supernatants of cells infected with lentivirus pseudotyped for spike protein, by immunoprecipitation assay. Three out of four mAbs lost their binding efficiency to completely N-deglycosylated rRBD and none was able to bind the same recombinant protein expressed in *Escherichia coli*, suggesting that the epitopes recognized by three mAbs are generated by the conformational structure of the glycosylated native protein. Of particular relevance, three mAbs were able to inhibit Wuhan SARS-CoV-2 infection of VERO E6 cells in a plaque-reduction neutralization test and the Wuhan SARS-CoV-2 as well as the Alpha, Beta, Gamma and Delta VOC in a pseudoviruses-based neutralization test. These mAbs represent important additional tools for diagnosis and therapy of COVID-19 and may contribute to the understanding of the functional structure of SARS-CoV-2 RBD.

## Introduction

An outbreak of severe transmittable pneumonia, later referred to as COVID-19 (COronaVIrus Disease 19), was first described in China in late 2019 (1). The disease resulted in high occurrences of fatal pneumonia with clinical symptoms resembling those of severe acute respiratory syndrome coronavirus (SARS-CoV) infections observed during the 2002-2004 SARS epidemic. Symptoms included persistent fever, chills/rigor, myalgia, malaise, dry cough, headache and dyspnea (2). The causative pathogen was identified as a novel coronavirus, initially designated 2019-nCoV and subsequently SARS-CoV-2 (3). SARS-CoV-2 has been able to spread rapidly worldwide since it is transmitted efficiently from human to human, even prior to the onset of symptoms, *via* droplets/aerosol from coughing or sneezing, or direct contact (4). In March of 2020, the World Health Organization officially declared COVID-19 as a pandemic. The emergence of virus variants of concern (VOC) with increased infectivity (Alpha, B.1.1.7; Beta, B.1.351; Gamma, P.1 and Delta, B.1.617.2) greatly contributed to the rise of infections (5) that, as of early October 2021 counted around 233,2 million confirmed cases with over 4.7 million deaths worldwide (https://covid19.who.int/). The pandemic is having a devastating impact on the global economy and public health systems worldwide. Therefore, in addition to safe and highly protective vaccines against SARS-CoV-2 and its VOC, monoclonal antibodies (mAbs), able to recognize and neutralize SARS-CoV-2 to be employed as new diagnostic tools and efficacious therapeutic approaches are still urgently needed.

SARS-CoV-2 is an enveloped virus with a positive single-stranded capped and polyadenylated RNA genome of about 30 kb. SARS-CoV-2 belongs to betacoronavirus genus in the *Coronaviridae* family. The genome has at least 10 open reading frames (ORF), ORF1a and ORF1b, produced by ribosomal frameshifting code for two long polyproteins, pp1a and pp1b, processed in 16 non-structural proteins (ns1-ns16) comprising the viral enzymes the RNA dependent RNA polymerase (RdRp) and two viral proteases (PL proteinase, 3CL). The non-structural proteins rearrange rough endoplasmic reticulum and Golgi compartments membranes into double-membrane vesicles where viral replication and transcription occur (viral factory). The entire replication cycle takes place in the cytoplasm. One-third of the genome encodes, in the order, four main structural proteins: spike (S), envelope (E), membrane (M) and nucleocapsid (N) proteins. Several small accessory proteins (ORF 3A,3B, 6, 7a, 7b, 8, 9a, 9b, 10) are coded in this region, some with important functions for the virus life cycle (6–8).

SARS-CoV-2 utilizes the transmembrane S glycoprotein to form homotrimers, protruding from the coronavirus particle surface, to mediate entry into host cells *via* the angiotensin-converting enzyme 2 (ACE2) receptor (9). The role of the Receptor Binding Domain (RBD) in the S protein suggests that immunization with this protein domain could induce antibodies (Abs) able to block virus binding and fusion thus neutralizing virus infection (10–12).

The RBD folds independently into a globular structure away from the rest of the S protein and exists in two different conformations as part of the trimer: “open” and “closed”. In the “open” state, it can bind ACE2, mostly by amino acid (aa) residues within a short segment called the Receptor Binding Motif (RBM). Many studies have shown that subunit protein antigens based on the RBD can elicit neutralizing antibodies (nAbs) against SARS-CoV (11–15). In this study, we produced SARS-CoV-2 recombinant RBD (rRBD) by expressing polyhistidine-tagged proteins in prokaryotic and eukaryotic systems and immunized mice with the protein produced in eukaryotic cells to induce RBD-specific antibody response. Spleen from mouse showing high humoral specific immune responses was isolated for the production of mAbs. We fully characterized four mAbs that showed a potent specific binding to the RBD and three of them were endowed with a strong capacity of neutralize SARS-CoV-2 and its VOC Alpha, Beta, Gamma and Delta.

## Materials and Methods

### Expression and Purification of rRBD

DNA sequence encoding the RBD (residue 318-538) was amplified by PCR from a mammalian cell codon optimized synthetic DNA sequence (Genescript Leiden, the Netherlands) encoding the ectodomain of SARS-CoV-2 spike protein Wuhan-Hu-1 isolate (NCBI Reference Sequence: NC_045512.2). Primer pair included an HindIII restriction site in the 5′ forward primer in frame with the murine Ig κ-chain leader sequence (5’-TTAAGCTTCAGGGTGCAGCCAACCGAGT-3’, HindIII site underlined) and an XhoI restriction site in the 3′ end reverse primer (5’-TTCTCGAGAGCACTTGTTCTTCACCAGATT-3’, XhoI site underlined) in frame with the polyhistidine tag of the plasmid pSecTag2HygroA (Invitrogen, Thermo Fisher Scientific) to produce pIgkRBD318-538.

Expression of rRBD was carried out in HEK293T cells grown in high glucose (4.5 g/l glucose) Advanced Dulbecco’s modified Eagle’s medium (Advanced DMEM, Thermo Fisher Scientific, MA, USA) supplemented with 10% fetal bovine serum (FBS, Gibco, Thermo Fisher Scientific), kanamycin (100 units/ml, Gibco) and 2 mM of L-glutamine (Gibco) incubated at 37°C and 5% CO_2_. Transfection was carried out using polyethylenimine (PEI) transfection reagent (Sigma). Briefly, cells were seeded overnight at 1x10^7^ cells in 175 cm^2^ flasks in 50 ml of Advanced DMEM containing 2% FBS. The culture medium was then removed and replaced with 45 ml of Ham’s F-12 Nutrient Mixture (F-12, Gibco) and transfected with 5 ml of Opti-MEM Reduced-Serum Medium (Gibco) containing 85 µg of DNA plasmid and 85 µl of PEI (0.5 mg/ml). Three days post-transfection, the culture supernatant was harvested, clarified by centrifugation, filtered and stored at 4°C. Protein purification was performed using Ni-NTA Agarose (Qiagen, Hilden, Germany) according to the manufacturer’s recommendations with minor modifications. Briefly, 100 ml of supernatant from rRBD-HEK293T transfected cells were combined to 100 ml of 2X Binding buffer (0.2 M sodium phosphate, 300 mM NaCl, 20 mM imidazole and 0.1% Tween 20, pH 8.0) and 1 ml of 50% slurry Ni-NTA agarose. The binding mix was incubated with gently agitation overnight at 4°C, loaded onto the column and ran by automated system. Column was washed with 100 ml of wash buffer (0.1 M sodium phosphate, 300 mM NaCl, 20 mM imidazole and 0.1% Tween 20, pH 8.0) and rRBD was eluted with 5 ml of elution buffer (0.1 M sodium phosphate, 300 mM NaCl, 250 mM imidazole and 0.05% Tween 20, pH 8.0) in ten different fractions. By SDS-PAGE analysis, fractions were selected then pooled and dialyzed in PBS buffer.

The theoretical mass of the HEK293T rRBD protein, deduced from the amino acid sequence, is 29.1 kDa.

For procaryotic expression in *Escherichia coli* (*E. coli*), DNA sequence encoding the RBD (residue 319-541) was amplified by PCR with the opportune primers (forward 5’-GCGCGGATCCAGGGTGCAGCCTACCGAATCAAT-3’ and reverse 5’-GCGCAAGCTTGAAGTTCACGCACTTGTTCTTGACC-3’) by using a codon optimized SARS-CoV-2 spike template DNA, obtained by *de novo* gene synthesis (Genescript) and cloned into BamHI/HindIII restriction sites of pQE30 (Qiagen). rRBD containing RGS(H)6 tag at N-terminus was purified by Ni-NTA (Qiagen) affinity chromatography using a denaturing protocol to optimize the yield and HisTRAP High Performance columns (Cytiva, Sweden AB). The theoretical mass of the *E. coli* rRBD protein, deduced from the amino acid sequence, is 26 kDa.

### SDS-PAGE and Western Blot (WB) Analysis

For the analysis of intracellular proteins, cell pellets were treated with 40 µl of cell extraction buffer (50 mM Tris-HCl, 250 mM NaCl, 1% NP-40; pH 7.4). Protein samples were lysed in SDS-loading buffer containing 50 mM Tris-HCl, pH 6.8, 3% SDS, 50% glycerol, 0.5% bromophenol blue, with or without 5% β-mercaptoethanol (βME). Samples were heated at 95°C for 5 min and loaded onto 7.5%, 4-20% or 4-15% gradient mini-PROTEAN TGX precast gels (Biorad). To monitor protein purification, gels were stained by SimplyBlueTMSafeStain (Novex, LifeTechnologies). For rRBD protein quantification gels were stained by fluorescent protein stain (Krypton Protein Stain, Thermo Scientific, USA) according to the manufacturer’s instructions using known amounts of bovine serum albumin (BSA) as standard. Gels were analyzed using ChemiDoc MP Imaging System (Biorad) and Image Lab Software (Image Lab 6.0.1). rRBD proteins resolved by SDS-PAGE were transferred onto polyvinylidene difluoride (PVDF) membrane (Thermo Fisher Scientific). The membranes were blocked with 3% skim milk in Tris-Buffer Saline and 0.05% Tween 20 (TBS-T) before incubation with anti-Tetra-His and anti-RGSHHHH mAbs (QIAGEN) for 1 h at room temperature. Immune complexes were detected with horseradish peroxidase (HRP)-conjugated goat anti-mouse immunoglobulin G (IgG) secondary antibody (Abcam, Cambridge, UK) for 1 h at room temperature. Signals were detected using either Crescendo Western HRP chemiluminescent substrate (Millipore, Burlington, MA, USA) or TMB colorimetric substrates (Vector, Burlingame, CA). SDS-PAGE in non-reducing condition was performed by resolving 100 ng of rRBD or virus infected VERO E6 cells (Cercopithecus aethiops derived epithelial kidney, ATCC CRL-1586) supernatant containing 1x10^6^ SARS-CoV-2 Plaque Forming Units (PFU)/ml denatured in SDS loading buffer without βME, followed by WB using anti-Tetra His (Qiagen) or anti-RBD mAbs.

### N-Deglycosylation of rRBD

N-deglycosylation of HEK293T-produced rRBD was performed using the peptide-N (4)-(N-acetyl-β-D-glucosaminyl) asparagine amidase F (PNGase F; Roche, Basel, Switzerland) according to manufacturer’s conditions. Briefly, rRBD was denatured for 10 min at 95°C, followed by the addition of PNGase F (1U/50 ng) and incubation a 37°C. Deglycosylated samples were diluted in SDS-loading buffer and incubated at 60°C for 15 min, then analyzed by SDS-PAGE followed by WB using anti-Tetra His mAb (Qiagen). In non-reducing reaction conditions, rRBD was treated with PNGase F in the presence of 2% SDS, diluted in SDS-loading buffer without βME and resolved in SDS-PAGE followed by WB using anti-His and anti-RBD mAbs.

### Plasmid Construction

Plasmid pSpike-C3 expressing the wild type codon optimized SARS-CoV-2 spike ORF (Wuhan-Hu-1, GenBank: NC_045512.2) containing a 21 aa deletion at the cytoplasmic tail (delta21) of the spike protein has been already described (16). Plasmid pSpike-FurPPC3 expresses the wild type codon optimized SARS-CoV-2 spike, contains a 21 aa deletion at the cytoplasmic tail (delta21) and was stabilized by the introduction of 2 prolines at aa positions 986 and 987 and by mutation at the furin site (RRAR into GSAS). pSpike-UKC3, pSpike-SAC3 and pSpike-BRC3 plasmids express the Alpha variant (lineage B.1.1.7), the Beta variant (lineage B.1.351) and the Gamma variant (lineage P.1) Spike ORFs, respectively, with a 21 aa deletion at the cytoplasmic tail. Plasmid pSpike-INd19 expresses the Delta variant (lineage B.1.617.2) spike ORF with a 19 aa deletion at the cytoplasmic tail. The Alpha variant B.1.1.7 pSpike-UKC3 used in these studies contained the following mutations: del69-70HV, del145Y, N501Y, A570D, D614G, P681H, T716I, S982A, D1118H. The Beta variant B.1.351 pSpike-SAC3 used in these studies contained the following mutations: L18F, D80A, D215G, del242-244LAL, R246I, K417N, E484K, N501Y, D614G, A701V. The Gamma variant P1 pSpike-BRC3 used in these studies contained the following mutations: L18F, T20N, P26S, D138Y, R190S, K417T, E484K, N501Y, D614G, H655Y, T1027I. The Delta variant B.1.617.2 pSpike-INd19 used in these studies contained the following mutations: T19R, del157-157, L452R, T484K, D614G, P681R, D950N.

For construction of pRetro-hACE2, a retroviral transfer vector expressing the human ACE2 receptor for SARS-CoV-2, a SpeI/BamHI fragment of DNA was removed from hACE2 plasmid (Addgene plasmid #1786) and inserted into XbaI/BamHI restriction sites of pQCXIN retroviral transfer vector plasmid (Clontech).

### Production of Lentiviral (LV) and Retroviral Vectors

293T Lenti-X cells (Clontech, Mountain View, CA, USA) were used for production of LV-Luc pseudotyped with Spike variants by transient transfection as previously described (16). Briefly, 293T Lenti-X cells (3.5x10^6^ cells) were seeded on 10 cm Petri dishes (Corning Incorporated - Life Sciences, Oneonta, NY, USA) and transiently transfected with plasmids pGAE-LucW, pADSIV3+ and the pseudotyping plasmid (pSpike-C3, pSpike-UKC3, pSpike-SAC3, pSpike-BRC3, pSpike-INd19 and control phCMV-VSV.G) using the JetPrime transfection kit (Polyplus Transfection Illkirch, France) following the manufacture’s recommendations using a 1:2:1 ratio (transfer vector: packaging plasmid: spike plasmid). At 48 h post transfections, culture supernatants containing the LV-Luc pseudotypes (LV-Luc/Spike-C3, LV-Luc/Spike-UKC3, LV-Luc/Spike-SAC3, LV-Luc/SpikeBRC3, LV-Luc/SpikeINd19 and LV-Luc/VSV.G) were collected and stored in 1 ml aliquots at -80°C until use.

Packaging Phoenix-AMPHO (ATCC) cell line was used for production of retroviral particles expressing hACE2 (Retro-hACE2). Briefly, cells were transiently transfected with the pRetro-hACE2 plasmid at 80% confluence in a 6 cm dish, by the calcium phosphate method. Cell medium (4 ml of DMEM, high glucose, Euroclone, supplemented with 10% FBS, glutamine and antibiotics) was replaced just before transfection, adding chloroquine (Sigma-Aldrich Merck KGaA) to a final concentration of 25 µM. Medium was further replaced 24 h after transfection and again 8 h later (this time with 2.5 ml only). After an additional 24 h, medium, containing retroviruses, was collected and centrifuged at 500 x g for 10 minutes (to pull down cells in suspension and cellular debris). After centrifugation, 2 ml of medium was carefully collected (to avoid disturbing the pellet) and used to transduce the murine melanoma cell line B16-F10 (ICLC, Genoa, Italy).

### Binding of rRBD to B16-F10 Cells Expressing hACE2

B16-F10 cells were transduced with Retro-hACE2 in the presence of 8 µg/ml Polybrene (Merck KGaA, Darmstadt, Germany). After 24 h of transduction, cells were seeded in B16-F10 growing medium after cell centrifugation at 400 x g for 10 min. Selection with 800 µg/ml of the antibiotic G418 (Sigma-Aldrich, St. Louis, MO, USA) was started 5 days later. After 5 passages, B16-hACE2 cells were incubated with mouse anti-hACE2 antibody (MAB5676-Millipore) followed by goat-anti-mouse PE (Biolegend) and hACE2 positive cells were sorted by FACSaria (Becton-Dickinson, Franklin Lakes, NJ, USA) and maintained with half dose of antibiotic. B16-F10 and B16-hACE2 cells were incubated with 100 µl of HEK293T rRBD (1 µg) for 30 min on ice. After three washes to remove unbound protein, the cells were incubated with the anti-Tetra-His mAb (Biorad) diluted 1:250 for 30 min on ice. Then, an anti-mouse IgG FITC-labeled, diluted 1:250 was added for 30 min on ice. After washes, the cell-associated fluorescence was measured by Beckman Coulter Gallios flow cytometer equipped with Kaluza Software (Beckman Coulter, Brea, CA, USA).

For blocking experiment, 1 µg of rRBD was preincubated or not with 2 µg/ml of anti-RBD mAbs for 30 min at 37°C in 100 µl of PBS+1%FBS. Then, the mixture was added to 50000 B16-F10 and B16-hACE2 cells for 30 min on ice. After 2 washes, 100 µl of anti-Tetra-His mouse mAb (Qiagen) diluted 1:250 was added and incubated for 30 min on ice followed by 30 min on ice incubation with an anti-mouse IgG FITC-labeled (Biolegend, diluted 1:250). After washes the cell-associated fluorescence was measured by Gallios cytometer (Beckman Coulter. Brea, CA, USA).

### Size Exclusion High-Performance Liquid Chromatography (SE-HPLC)

The chromatographic analysis of rRBD was performed with an Alliance Waters e2695 HPLC system (Waters Corporation, Milford, Massachusetts) controlled by the Empower software. SE-HPLC analysis was performed isocratically (with a constant concentration of the mobile phase) at room temperature using a Tosoh bioscience guard-column TSK gel SWXL (40 × 6 mm) and a Tosoh bioscience HPLC column TSK gel G3000SWXL (300 x 7.8 mm, 5 µm). The mobile phase consisted of 100 mM phosphate buffer with 100 mM Na_2_SO_4_, at pH 7.2. The flow rate was 0.5 ml/min and the total analysis time was 35 min. The protein was injected without any dilution/pretreatment and the injection volume was 175 μl. A Bio-Rad’s Gel Filtration Standard (mixture of molecular weight markers ranging from 1,350 to 670,000 Da) was used as calibrator. The elution was monitored with a PDA 2998 Waters set at 214 nm (17).

### Mice Immunization

Six-week-old female pathogen-free BALB/c mice were obtained from Charles River Laboratories (Calco, LC, Italy) and housed in the Istituto Superiore di Sanità. All animal protocols and procedures were performed in accordance with European Union guidelines and Italian legislation (DL26/2014) and have been approved by the Italian Ministry of Healthy and reviewed by the Service for Animal Welfare at ISS (Protocol n. 670/2020-PR of July 21^st^, 2020). Mice were immunized with a subcutaneous injection on both sides of lower anterior abdomen on days 0, 14 and 28. Five µg of HEK293T rRBD in 50 µl were mixed with an equal volume of emulsified complete Freund’s adjuvant (Millipore-Sigma) for priming or with incomplete Freund’s adjuvant (Millipore-Sigma) for boosts respectively, immediately prior to administration. Blood samples were collected on days 0 (pre-immunization) and at day 42 (2 weeks after the third immunization) by retro-orbital collection, centrifuged and sera stored at -20°C until analyzed. The collected sera were tested by *in-house* ELISA for the determination of anti-RBD specific antibody titres in order to identify the high responder mouse. The selected mouse received a final boost of 5 μg of HEK293T rRBD in the absence of adjuvant by intravenous injection. Three days later the mouse was sacrificed, splenocytes were prepared by mechanical disruption and passage through cell strainers (BD Pharmingen, San Diego, CA, USA) and resuspended in RPMI 1640 (Euroclone) containing 10% FBS (Gibco), 100 units/ml of kanamycin (Gibco), non-essential aminoacids (Gibco), 1 mM sodium pyruvate (Gibco) (complete RPMI medium) with the addition of 25 mM Hepes buffer solution (Euroclone).

### Hybridoma Fusion and Isolation of Specific mAb-Producing Clones

Mouse myeloma cell line SP2 (ATCC. Manassas, VA, USA) was cultured in complete RPMI medium. Spleen cells isolated from the selected mouse were incubated with 5 ml lysis buffer on ice for 5 min. After washing, splenocytes were resuspended into serum-free RPMI supplemented with 25 mM Hepes and counted. SP2 cells were then mixed with the splenocytes, centrifuged at 1000 rpm for 10 min and slowly resuspended with 1 ml of polyethilenglycol (PEG, Sigma-Aldrich). Then, 7 ml of complete RPMI medium supplemented with 25 mM of Hepes was slowly added, the cells centrifuged, and the pellet resuspended in complete RPMI medium containing hypoxanthine, aminopterin and thymidine (HAT, Sigma-Aldrich). After fusion, cells were cultured in 96 well plate flat bottom (Corning) for 14 days at 37°C with 5% CO_2_ and the resultant fused growing cell lines were selected by microscope examination, transferred to individual wells in new 96-well plates and expanded in complete RPMI with HAT. Hybridomas were screened for antigen specificity of produced Abs by ELISA. The selected polyclonal Ab-producing hybridomas were single-cell-cloned by limiting dilution in the presence of 5x10^4^ cells/well feeder splenocytes in 96 well flat bottom plate in complete RPMI medium with HAT for 12 days. The growing clones were tested for antigen specificity by ELISA and those producing mAbs specific for RBD expanded in static T75 flasks (Corning) in serum free medium DCCM2 (Biological Industries) supplemented with kanamycin, hypoxanthine and thymidine (HT, Sigma-Aldrich).

### Monoclonal Antibody Purification

The clones producing mAbs specific for SARS-CoV-2 RBD were expanded and the mAbs purified and concentrated by using chromatography cartridges protein G columns (Thermo Fisher). Briefly, the protein G column was washed with PBS, then 30 ml of mAb culture supernatant was diluted 1:1 in PBS and passed over the column. Finally, the bound mAb was eluted with 0.1 M glycine pH 2.5 and the pH immediately neutralized with 1 M Tris pH 7.9 before dialysis with PBS. The concentration of purified mAbs was evaluated by NanoPhotometer (Implen, Münich, DE) spectrophotometer at 280 nm and expressed as µg/ml.

### Human Sera

Pseudonymized sera from healthy or COVID-19 convalescent volunteers who gave their informed consent to donate blood for research purposes and to participate to the collaborative study between Istituto Superiore di Sanità and Italian Air Force entitled: “*Valutazione della performance analitica di un test antigenico per il rilevamento di SARS-CoV-2, confronto con un test di screening molecolare*” were heat-inactivated at 56°C for 30 minutes and frozen until use.

### ELISA

rRBD was plated o/n at 4°C on high binding flat-bottom 96-well polystyrene plates (Greiner Bio-One, Rainbach, Austria) at 0.5 μg/ml (50 µl/well) in sodium bicarbonate (pH 9.0) buffer. Then plates were washed with PBS+0.5% Tween 20 and incubated with 200 µl/well of a blocking solution (postcoat) of PBS containing 2% BSA (w/v) for 1 h at RT. Then, 50 µl of serum or hybridoma supernatants diluted in blocking buffer were incubated for 3 h at 37°C, followed by washing. Goat anti-mouse IgG alkaline phosphatase (PA)-conjugated (Southern Biotech, Birmingham, AL, USA) or mouse anti-human IgG PA-conjugated (Invitrogen) (1:1000 in blocking buffer) were incubated for 1 h at 37°C. After washes, PA substrate (Sigma) was added, the enzymatic reaction stopped by 3N NaOH and absorbance (405 nm) measured by Varioskan Flash reader (Thermo Fisher Scientific). Results were considered positive when the optical density (OD) obtained with the mAbs was three times greater than the negative control. The subclass of isolated mAbs was identified using enzyme-conjugated anti-mouse subclass specific antibody (anti-IgG1, IgG2a, IgG2b, IgG3; Southern Biotech)

For competitive ELISA, plate was coated with 0.5 µg/ml of HEK293T rRBD o/n and after 1 h of incubation with the postcoat solution, 50 µl/well of a mixture of fixed R590 and R64 mAbs concentration (0.02 µg/ml) and two-fold dilutions (starting form 1:25) of human sera were added and incubated 3 h at 37°C. Sera from convalescent COVID-19 patients with known titers of neutralizing antibodies (nAbs) and sera from healthy subjects were tested. Then, goat anti-mouse IgG PA-conjugated antibody (1:1000; Southern Biotech) was added for 1 h at 37°C, followed by development of enzymatic reaction.

### Evaluation of the mAbs Binding Capacity by Flow Cytometry

To analyze the binding of anti-RBD mAbs to the native trimeric spike, HEK293T cells transfected with pSpike plasmids expressing the transmembrane wild-type S protein of SARS-CoV-2 Wuhan or VOC were collected by trypsinization. After washing, 1x10^5^ cells per polypropylene tube were incubated for 30 min on ice with 100 µl of anti-RBD mAbs at serial concentrations (starting from 0.4 µg/ml) diluted with PBS+1% FBS or with murine IgG1 negative control mAb (SinoBiological, USA). Anti-S2 (40590-T62 SinoBiological, USA) antibody was used as a positive control of spike expression, as described above. After two washes to remove unbound mAbs, a goat anti-mouse IgG FITC-labeled (Southern Biotech, Birmingham, AL, USA) mAb was added (dilution 1:250) and incubated 30 minutes on ice. Rabbit anti-S2 (40590-T62 SinoBiological, USA) antibody followed by a donkey anti-rabbit FITC-labeled mAb (Biolegend, San Diego, CA, USA) was used as a positive control of spike expression. After two washes, the cells were resuspended with PBS+1%FBS and their associated fluorescence measured by flow cytometry by acquiring 2x10^4^ events in a large forward and side scatter-based gate by using a Beckman Coulter Gallios with Kaluza Software (Beckman Coulter).

### Immunoprecipitation (IP)

Immunoprecipitation of LV pseudotyped with pSpike-FurPPC3 (LV-FurPPC3) or with VSV.G (LV-VSV.G) as negative control was performed using Dynabeads Protein G (Thermo Fisher), according to the manufacturer’s instructions. Briefly, purified mAbs R590 and R64 were conjugated to protein G beads for 30 min at room temperature. Beads were washed twice in IP buffer (20 mM Tris-HCl pH 7.4, 150 mM NaCl, 0.1% NP-40) and incubated with Lenti-X cell supernatant containing pseudotyped LV in IP buffer for 2 hours at room temperature. After washing three times in IP buffer, beads were eluted with SDS-loading buffer at 95°C for 10 min. Immunoprecipitation was analyzed by SDS-PAGE followed by WB using an anti-S1 mAb (MA5-36247, Thermo Fischer).

### Pseudovirus Titration and Neutralization Assay

Preparations of LV-Luc Wuhan, Alpha, Beta, Gamma and Delta pseudotypes (LV-Luc/Spike-C3, LV-Luc/Spike-UKC3, LV-Luc/Spike-SAC3, LV-Luc/SpikeBRC3 and LV-Luc/SpikeINd19) were titered in VERO E6 cells seeded in a 96-well plate (View plate, PerkinElmer) at a density of 20000 cells/well. After 48 h, luciferase expression was determined by the britelite plus Reporter Gene Assay System (PerkinElmer) and measured with a Varioskan luminometer (Thermo Fisher). Dilutions providing 150000-200000 relative luminescence units (RLU) were used in the neutralization assay. Briefly, mAb serial 2-fold dilutions starting from 20 µg/ml were incubated in duplicate with the Wuhan, Alpha, Beta, Gamma and Delta LV-Luc pseudoviruses for 30 min at 37°C in 96-deep well plates (Resnova, Genzano di Roma, Italy), and thereafter added to VERO E6 cells seeded in a 96-well View plate at a density of 20000 cells/well. Virus-only and cell-only controls were included. After 48 h, luciferase expression was determined by the britelite plus Reporter Gene Assay System (PerkinElmer). RLU data points were converted to a percentage neutralization value, calculated relative to virus-only controls. Results are expressed as inhibitory concentration (IC) 50 corresponding to the mAb concentration giving 50% inhibition of infection (neutralization) compared to the virus control wells (16).

### Virus Propagation

VERO E6 cells were grown in Dulbecco’s modified Eagle’s medium (DMEM, Gibco) supplemented with 2.5% heat-inactivated Fetal Calf Serum (FCS), 100 units/ml penicillin, 100 μg/ml streptomycin, 2 mM L-glutamine, 1 mM sodium pyruvate, and 1x non-essential amino acids (Gibco).

Viral isolate BetaCov/Italy/CDG1/2020|EPI ISL 412973|2020-02-20 (GISAID accession ID: EPI_ISL_412973) was propagated by inoculation of 70% confluent VERO E6 cells in 75 cm^2^ cell culture flasks. Cells were observed for cytopathic effect every 24 h. Stocks of SARS-CoV-2 virus were harvested at 72 h post infection, and supernatants were collected, clarified, aliquoted, and stored at -80°C. Infectious virus titer was determined as PFU. For some experiments of WB and IP, SARS-CoV-2 virus was inactivated at 56°C for 30 minutes.

### Plaque Reduction Neutralization Test (PRNT)

Method used for PRNT was essentially the same as previously described (18). The viral stocks of SARS-CoV-2 were titred three times by semi-log10 dilutions by plaque forming assay. Then serial 2-fold dilutions (starting from 5µg/ml) of purified mAbs were incubated with 80 PFU of SARS-CoV-2 at the final volume of 600 μl at 4°C overnight. The mixtures were added in duplicates to confluent monolayers of VERO E6 cells, grown in 12-well plates and incubated at 37°C in a humidified 5% CO_2_ atmosphere for 60 min. Then, 4 ml/well of a medium containing 2% Gum Tragacanth (Sigma Aldrich) + MEM 2.5% FCS were added. Plates were left at 37°C with 5% CO_2_. After 3 days, the overlay was removed, and the cell monolayers were washed with PBS to completely remove the overlay medium. Cells were stained with a crystal violet 1.5% alcoholic solution. The presence of SARS-CoV-2 virus–infected cells was indicated by the formation of plaques. The IC50 was determined as the highest dilution of serum resulting in 50% (PRNT_50_) reduction of plaques as compared to the virus control.

### Statistical Analysis

For neutralization and ELISA experiments technical duplicates or triplicates were performed. All of the statistical analyses were performed using the GraphPad Prism software v9 (GraphPad Software, San Diego, CA, USA) (16). The IC50 were calculated with a non-linear regression method. The EC_50_ were calculated by non-linear regression analysis of log10 of serum dilution plotted versus absorbance at 405 nm. Statistical analysis of data in figure 2A and 3A were performed using the non-parametric Mann-Whitney 2-sided *U*-test and the Kruskal-Wallis multiple comparison test. The *p* values < 0.05 were considered significant.

## Results

### Characteristics of the Produced rRBD

**Figure 1:**
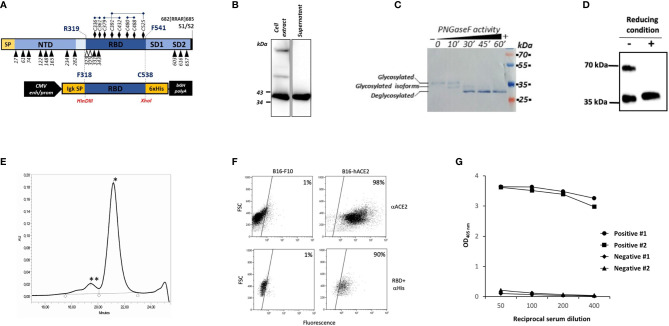
Production and characterization of HEK293T rRBD. **(A)** Diagram of SARS-CoV-2 S1 domain and of the rRBD expression cassette in pIgkRBD_318-538_ SARS-CoV-2 plasmid vector. Functional elements are indicated: SP (signal peptide), NTD (N-terminal domain), RBD, SD1 and SD2 (subdomain 1 and 2), CMV enh/prom (human cytomegalovirus immediate early enhancer/promoter), Igk SP (murine Ig κ-chain leader sequence), 6xHis (polyhistidine tag) and bGH polyA (bovine growth hormone polyadenylation signal). N-glycosylation and O-glycosylation sites are indicated by black and white respectively triangles. Cysteine residues are indicated by black diamond and disulfide bonds are reported. **(B–D)** WB characterization of rRBD using an anti-Tetra His mAb. **(B)** Detection of rRBD in the cell extract and in the supernatant of transfected HEK293T cells. **(C)** Purified rRBD was denatured and digested with PNGase F in a time-dependent manner. The positions of glycosylated, deglycosylated and glycosylated isoforms of rRBD are indicated. **(D)** rRBD was denatured in SDS-loading buffer without βME or with βME. **(E)** SE-HPLC chromatogram of rRBD. The retention time suggests an approximate molecular mass of less than ∼40 kDa (*) corresponding to the monomeric form and another molecular mass of >150 kDa (**) corresponding to the aggregate multimeric structure. **(F)** rRBD binding to ACE2 receptor by flow cytometry. In the upper panels B16-F10 or B16 cells expressing human ACE2 receptor (B16-hACE2) were stained with anti-ACE2 mouse mAb and goat anti-mouse IgG PE-conjugated as secondary antibody. In the lower panel cells were incubated with rRBD and then stained with an anti-histidine mouse mAb revealed with goat anti-mouse FITC-conjugated mAb. The numbers indicate the percentages of positive cells. Each panel from **(B–F)** shows a representative experiment repeated at least two times. **(G)** Binding of serial dilution of human serum from two COVID-19 positive and two negative subjects on rRBD coated to the ELISA plate. Graph reports results obtained by two out of seven positive and seven negative subjects.

Based on the solved structure provided by Walls et al. (9), the sequence of the RBD covering amino acids from F318 to C538 (**Figure 1A**) was selected for expression of a secreted His-tagged protein from human HEK293T cells and then analyzed by SDS-PAGE and WB. As shown in figure 1B, rRBD was well detected in both the cell lysate and the supernatant of transiently transfected HEK293T cells as a band of approximatively 35 kDa. This result contrasts with the predicted molecular mass of 29.1 kDa calculated on the basis of rRBD amino acid sequence but could be explained by post-translational modifications that occur in mammalian cells. Indeed, two N-glycosylations and one O-glycosylation have been described at SARS-CoV-2 RBD N-terminal region (19) (**Figure 1B**). To verify the role of N-glycosylation on rRBD electrophoretic mobility, we analyzed by WB the molecular weight of rRBD before and after the enzymatic treatment with the glydosidase PNGase F. The time-dependent decrease of the observed rRBD molecular mass in correlation with PNGase F exposure, confirmed the presence of N-linked glycosylations in the rRBD (**Figure 1C**).

To evaluate the role of cysteine residues in disulfide bonds formation and the potential presence of RBD multimeric structures, we compared the mobility of rRBD on SDS-PAGE under reducing and non-reducing conditions. Notably, rRBD in the absence of reducing agents ran as two different bands (**Figure 1D**), as previously observed by Farnos et al. (20). This data confirms that the unfolded state of rRBD is constrained by the native disulfide bonds and indicates that in the folded state multimeric structures can be formed (20). This conclusion is supported by SE-HPLC analysis showing that purified rRBD exists mainly as a monomer, with a small fraction of rRBD producing aggregates, as shown by the presence of two different peaks in the chromatogram (**Figure 1E**). The rRBD preparations used for all the experiments were checked for quantitative analysis by Krypton stained SDS-PAGE that always showed the absence of contaminants (**Supplementary Figure S1**)

We next evaluated the ability of rRBD to bind the hACE2 receptor on B16-hACE2 cells, the B16-F10 murine cell line stably transduced with hACE2, as a measure for assessing the structural integrity and the correct folding of the protein. An anti-hACE2 mAb confirmed that hACE2 was expressed on the surface of B16-hACE2 cells (**Figure 1F**) and we could show that rRBD binds to B16-hACE2 cells but not to control B16-F10 cells, demostrating that the produced recombinant protein maintains the functional properties of the viral RBD, namely its capacity to bind human ACE2.

Finally, we used previously screened sera from normal controls or COVID-19 convalescent individuals with known levels of anti-RBD specific Abs to test the antigenic properties of produced recombinant protein. Only sera from patients with anti-SARS-CoV-2 Abs bound to rRBD (**Figure 1G**). These results confirm that our purified recombinant protein retains native structure and preserves the antigenic properties of the SARS-CoV-2 spike RBD.

### Binding Characteristics of RBD Specific mAbs

**Figure 2:**
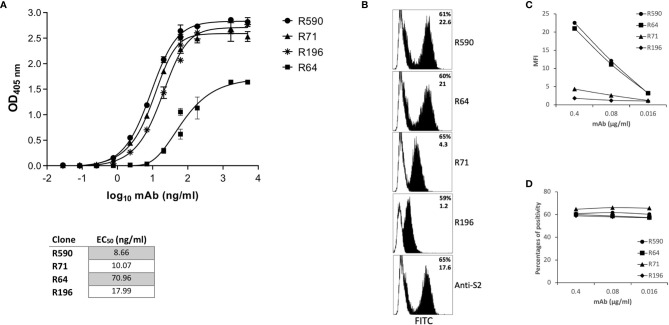
Characterization of anti-RBD mAbs. **(A)** ELISA-binding affinity of purified mAbs to rRBD produced in mammalian expression system. Error bars indicate standard deviations of technical triplicates from a representative experiment repeated twice. Table indicates the EC_50_ of isolated mAbs. **(B–D)** HEK293T cells transfected with plasmid coding for wild type transmembrane homotrimeric spike (pSpike-C3) were incubated with anti-RBD mAbs at 0.4 µg/ml (**B**, black histograms) or with murine IgG1 control mAb (**B**, white histograms) or with serial dilution of mAbs starting from 0.4 µg/ml **(C, D)** followed by staining with anti-mouse FITC-conjugated mAb before analysis by flow cytometry. An anti-S2 mAb was used as control to determine the percentage of transfection. In **(B)** the upper numbers indicate the percentages of positive cells, lower numbers indicate mean fluorescence intensity. In **(C)** mean fluorescence intensity (MFI) of staining is shown. In **(D)** percentages of positive stained cells is shown. A representative experiment of three is shown.

Mice were immunized with rRBD expressed in mammalian HEK293T cells to generate specific Abs. Sera from immunized mice were screened for RBD binding by ELISA, and the mouse with the highest titers of serum-specific IgG was sacrificed and splenocytes were fused with a mouse myeloma. Hybridomas secreting anti-RBD Ab were cloned by limiting dilutions to obtain monoclonal cultures. Four monoclonal hybridomas were selected and the secreted mAbs R64, R71, R196 and R590 were purified on protein G columns from the culture supernatant and their chracteristics were tested by ELISA, flow cytometry, WB, IP and functional assays. The selected mAbs were IgG1 (data not shown) and bound HEK293T rRBD in the ELISA (**Figure 2A**). The half maximal effective concentration (EC_50_) required for all mAbs to bind HEK293T rRBD glycoprotein falls below 100 ng/ml (**Figure 2A**): in particular R590, R71 and R196 were characterized by high binding affinity (EC_50_: 8.66, 10.07 and 17.99 ng/ml respectively), while R64 shows the lowest affinity (EC_50_: 70.96 ng/ml).

We confirmed the mAb ability to bind RBD also when this protein domain is part of the homotrimeric spike complex exposed on the surface of transfected HEK293T cells (**Figure 2B**). An anti-S2 mAb was used to determine the percentage of fully transfected HEK293T cells. As shown in figure 2B, the four mAbs recognized the trimeric spike glycoprotein (**Figure 2B**) showing a different efficiency of binding in terms of mean fluorescence intensity (**Figure 2C**): R590=R64>R71>R196. Interestingly, at low doses the mAbs were still able to stain all the transfected cells (**Figure 2D**).

### mAbs Recognize Conformational Epitopes of the Glycosylated RBD

**Figure 3:**
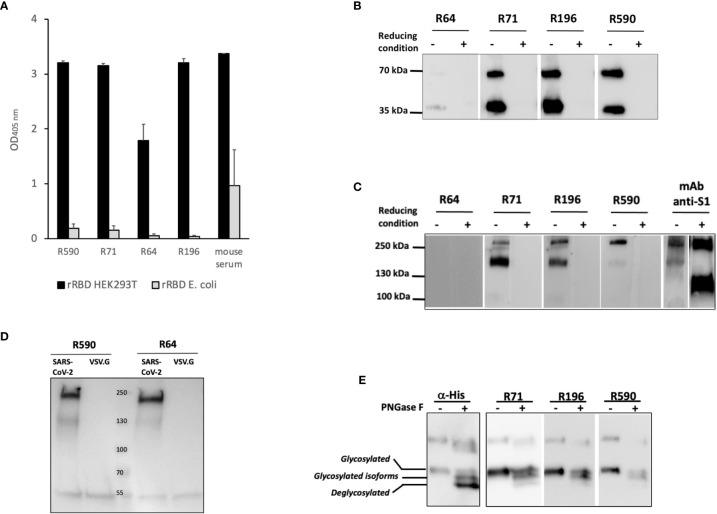
Binding of mAbs to RBD requires disulfide bonds and glycosylation. **(A)** Binding of mAbs to rRBD produced in mammalian (RBD HEK) or prokaryotic (RBD *E. coli*) expression system evaluated by ELISA. Serum from a mouse immunized with rRBD HEK was used as positive control of antigenicity. Error bars indicate standard deviations of technical duplicates from a representative experiment repeated three times. **(B, C)** rRBD **(B)** or VERO E6 cell supernatant containing 1x10^6^ SARS-CoV-2 PFU/ml **(C)** were denatured without (non-reducing condition) or with (reducing condition) βME and resolved on SDS-PAGE followed by WB using R64, R71, R196 and R590 mAbs. Anti-S1 mAb was used as positive control. **(D)** HEK293T cell supernatant containing lentiviral vector pseudotyped with pSpike-FurPPC3 (SARS-CoV-2) was incubated with mAbs R590 and R64 conjugated to protein G. Lentiviral vector pseudotyped with VSV.G (VSV.G) was used as negative control. Immunoprecipitation was analyzed by SDS-PAGE followed by WB using anti-S1 mAb. **(E)** rRBD N-deglycosylation was performed in denatured, non-reducing conditions and revealed using α-His mAb and R71, R196 and R590. The positions of glycosylated, deglycosylated and glycosylated isoforms of rRBD are reported. Each panel from **(B–E)** shows a representative experiment repeated at least two times.

In the attempt to characterize the epitopes recognized by our mAbs, we first compared their reactivity to the rRBD produced in HEK293T or to rRBD produced in a prokaryotic expression system. To this end, the RBD domain was produced in *E. coli* as His-tagged fusion protein that ran as a ∼26 kDa single band in WB and Coomassie staining (**Supplementary Figure S2**). This prokaryotic expression system is normally inefficient in disulfide bonds formation when recombinant proteins are expressed in the cytoplasm as inclusion bodies (21) that require denaturing agents for solubilization. Indeed, the produced *E. coli* rRBD ran as a single band in both reducing and non-reducing conditions (data not shown). Sera of immunized mice were able to bind both the HEK293T and *E. coli* rRBD proteins in ELISA, although the rRBD produced in *E. coli* was recognized with low efficiency. On the contrary, all the tested mAbs were shown to bind the HEK293T rRBD, but not the *E. coli* rRBD (**Figure 3A**). Interestingly, none of our mAbs recognized the rRBD produced in both HEK293T and *E. coli* expression systems in WB under reducing and denaturing conditions (data not shown). These data suggest that immunization with HEK293T rRBD results in a small fraction of antibody recognizing linear epitopes and a larger array of IgG, which includes our mAbs, that are likely to recognize discontinuous epitopes (22). To evaluate the role of disulfide bonds in the generation of conformational epitopes, we tested the reactivity of our mAbs to the HEK293T rRBD in WB following SDS-PAGE under either reducing or non-reducing conditions (**Figure 3B**). As expected, none of mAbs recognized the rRBD in reducing conditions, but mAbs R71, R196, R590, and to a lesser extent R64, bound HEK293T rRBD in the absence of reducing agents. The role of disulfide bonds in the generation of the relevant epitopes was confirmed by the observation that R71, R196 and R590, but not R64, recognized both the full-length spike and the cleaved S1 domain present in the heat inactivated supernatant of SARS-CoV-2 infected VERO E6 cells only in non-reducing conditions (**Figure 3C**). To ascertain whether the absence of binding with mAb R64 could be ascribed to alteration of protein conformation due to the experimental conditions that included the use of SDS (23), we performed an immunoprecipitation assay using LV pseudotyped with SARS-CoV-2 spike protein. As shown in **Figure 3D**, R64 as well as R590, used as a positive control, were able to immunoprecipitate the SARS-CoV-2 pseudovirus, but not the VSV.G psudotyped LV, indicating that R64 binds RBD of SARS-CoV-2 in its native, unmodified conformation.

To better characterize the binding of mAbs to HEK293T rRBD, we analyzed the involvement of glycosylation. N-glycosylations have been described on residues N-331 and N-343 of the spike RBD (19) and we have shown (**Figure 1C**) that HEK293T rRBD shares the co-translational N-glycosylation described for the SARS-CoV-2 spike protein. **Figure 3E** shows that R71, R196 and R590 lose the binding efficiency to the fully de-glycosylated form of rRBD, indicating that these mAbs do not bind linear, but conformational epitopes of the glycosylated protein.

### Three mAbs Show SARS-CoV-2 Neutralization Capacity

**Figure 4:**
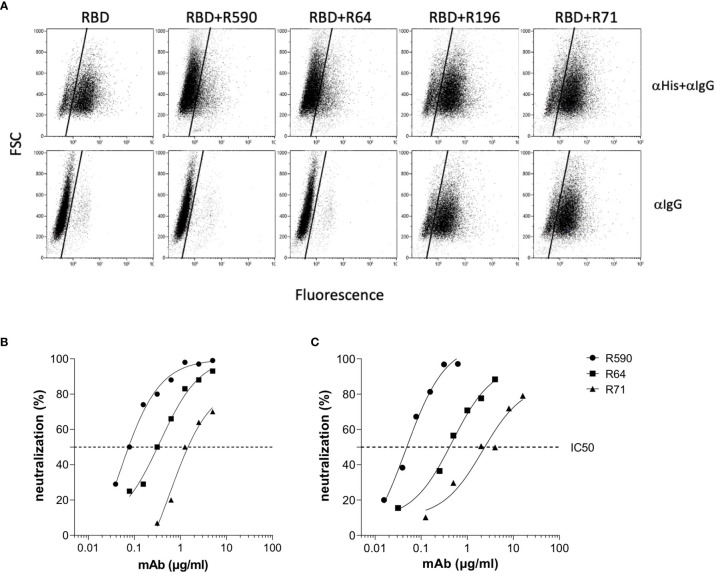
mAb inhibition of RBD-ACE2 binding and neutralization activity against Wuhan SARS-CoV-2. **(A)** B16-hACE2 cells were incubated with rRBD alone or with rRBD pre-incubated with isolated mAbs. In the upper panels the ACE2-RBD binding was quantified by measuring the signal given by an α-His mAb revealed with a FITC-conjugated α-mouse IgG mAb (αHis+αIgG). In the lower panels, after rRBD incubation, an α-mouse IgG FITC-conjugated mAb was added in order to reveal anti-RBD mAbs bound to the hACE2 receptor. A representative experiment of two is shown. **(B)** Neutralization curves of anti-RBD mAbs against SARS-CoV-2 by PRNT or **(C)** spike pseudotyped LV neutralization assay. Data are representative of one experiment out of three with two technical replicates.

The selected mAbs were evaluated for their ability to prevent the binding of RBD to its receptor ACE2 by using B16-hACE2 cells in a flow cytometry blocking experiment. Pre-incubation of rRBD with mAbs R590 or R64 showed a drastic reduction of its binding to ACE2 receptor (**Figure 4A**, upper panels). This binding inhibition was not observed after the pre-incubation with R196 and R71 even if RBD-mAbs immunocomplexes were bound to the receptor as shown using anti-mouse IgG mAb (**Figure 4A**, lower panels), proving that these mAbs bind epitopes outside the RBM and not involved in ACE2 interaction.

The ability of our mAbs to neutralize SARS-CoV-2 was evaluated using two experimental approaches. The neutralization potential against the authentic virus was evaluated by PRNT using SARS-CoV-2 isolated from COVID-19 patients. mAbs R590 and R64, and to a lesser extent R71, were endowed with a strong neutralization capacity that correlated with the efficiency of binding to the native protein on cell surface, while R196 was not neutralizing. R590 showed the highest potency against the authentic virus (IC50: 0.08 µg/ml) followed by R64, while R71 showed limited neutralizing capacity (IC50 = 0.312 and 1.25 µg/ml, respectively) (**Figure 4B**, left panel). In contrast, the mAb R196, did not show any virus neutralization under these experimental conditions (data not shown). The neutralizing activity of the mAbs was also evaluated using a pseudovirus neutralization assay, based on LV expressing Luciferase and pseudotyped with SARS-CoV-2 spike (LV-Luc/Spike) (16) (**Figure 4B**, right panel). As expected, the IC50 values are slightly different from those calculated by a PRNT assay; however, the relative neutralizing potency among the mAbs was confirmed. Indeed, R590 was the most potent nAb with the lowest IC50 compared to R64 and R71 (IC50 = 0.045, 0.474 and 2.157 µg/ml, respectively), while R196 mAb was not able to neutralize the pseudovirus (data not shown).

### The mAbs R590, R64 and R71 Bind SARS-CoV-2 Trimeric Spike Belonging to Major VOC and Neutralize the Corresponding Pseudoviruses

**Figure 5:**
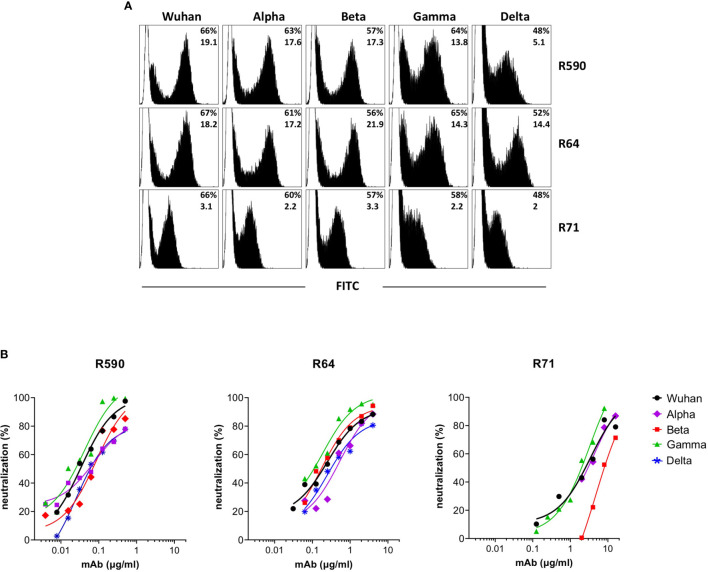
Binding and neutralization potency of anti-RBD mAbs against Wuhan and VOC. **(A)** HEK293T cells transfected with plasmid coding for wild type transmembrane homotrimeric spike of Wuhan or VOC were stained with R590, R71, R64 neutralizing anti-RBD mAbs (black histograms), or with murine IgG1 control mAb (white histograms) followed by goat anti-mouse FITC-conjugated mAb and analysed by flow cytometry. The upper numbers indicate the percentages of positive cells, lower numbers indicate mean fluorescence intensity. A representative experiment is shown. **(B)** Neutralization curves obtained by using LV pseudotyped with spike of Wuhan or its Alpha, Beta, Gamma and Delta VOC testing serial dilutions of isolated R590, R64 and R71 mAbs. Data are mean representative of three independent experiments each with two technical replicates.

With the worldwide progression of COVID-19 pandemic, several new SARS-CoV-2 variants containing mutations in the spike protein have been isolated, showing increased infectivity and ability to cause disease in susceptible individuals. Clinical studies were designed to define whether immunization with vaccines based on the original SARS-CoV-2 Wuhan-Hu-1 spike sequence may be sufficiently protective against these VOC (24–26). Moreover, mAbs developed for diagnostic or therapeutic purposes may not be useful for individuals infected with these variants and/or affected by COVID-19 (27). First, we measured the ability of the neutralizing RBD-specific R590, R71 and R64 mAbs to bind their epitopes expressed on the surface of HEK293T cells transfected with plasmids coding for the trimeric spike with the original (Wuhan-Hu-1) protein sequence and with the sequences of the Alpha, Beta, Gamma and Delta variants. The three SARS-CoV-2 nAbs recognized the original native spike protein as well as the four main variants (**Figure 5A**). Importantly, the mAbs R590, R64 and R71 inhibited the infection of VERO E6 cells with all the variant strains in the pseudovirus neutralization assay (**Figure 5B**), indicating a broad neutralizing activity of these mAbs.

### Sera of Patients With nAbs Generated During Natural SARS-CoV-2 Infection Compete for the Same Epitopes Recognized by Our Neutralizing mAbs

**Figure 6:**
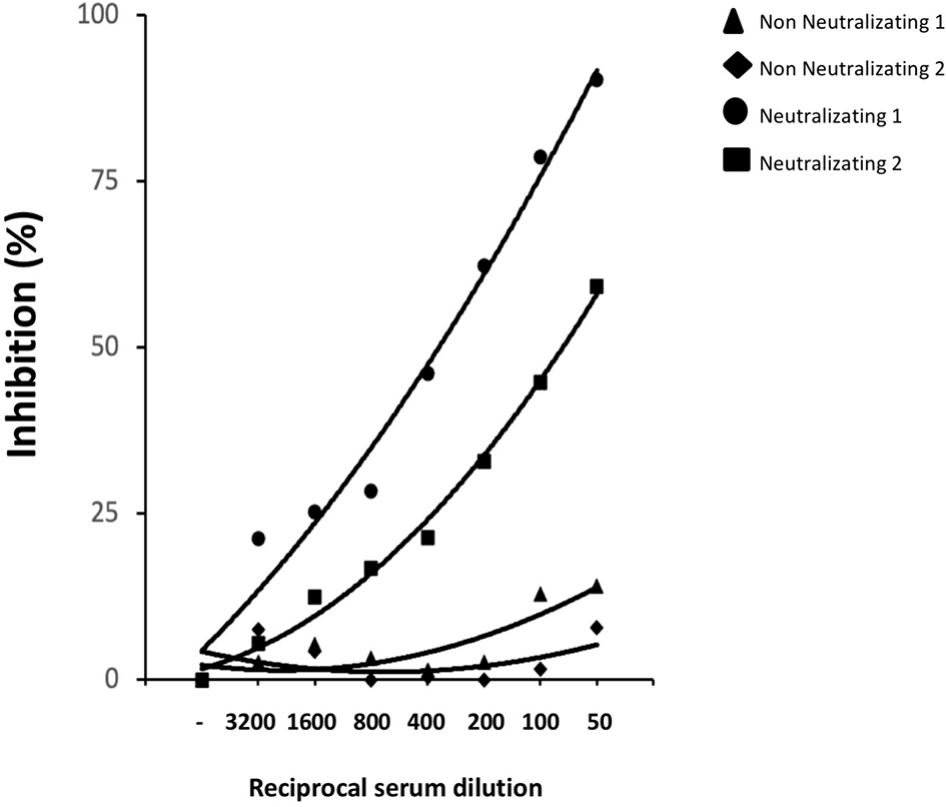
Competition immune-enzymatic assay. Increasing amounts of human serum from two COVID-19 patients with nAbs or two healthy subjects were mixed with a fixed amount of neutralizing R590 and R64 mAbs to perform a competitive ELISA. The reduction of the mAbs binding to HEK293T rRBD was measured as absorbance reduction and considered dependent on nAb concentration in sera. Results are expressed as percentage of inhibition compared to the maximum binding of R590 and R64 in the absence of the human anti-RBD neutralizing serum as competitor. Results from two representative neutralizing sera out seven and two non-neutralizing out of six are shown.

We asked whether the same conformation and glycosylated structure of the epitopes recognized by our neutralizing mAbs may be relevant for the *in vivo* generation of nAbs in SARS-CoV-2 infected individuals. We selected sera from COVID-19 convalescent patients showing high SARS-CoV-2 neutralizing capacity or from individuals with no measurable neutralizing activity to test whether these sera were able to compete for the same neutralizing epitope(s) recognized by mAbs R590 and R64. **Figure 6** shows that increasing concentration of neutralizing sera from COVID-19 patients progressively reduced the binding of the mixture of mAbs R590 and R64 to coated rRBD, while non neutralizing sera were ineffective to prevent the binding of our nAbs to rRBD. These data indicate that convalescent COVID-19 patients developed nAbs that compete for the same epitopes recognized by our neutralizing mAbs, suggesting that conformational and epitopes of the glycosylated RBD are crucial for SARS-CoV-2 infectivity.

## Discussion

An unprecedented global effort to counteract the COVID-19 pandemic started in early 2020 to identify appropriate public health strategies and to develop drugs, vaccines and nAbs against SARS-CoV-2 (28, 29). COVID-19 is still a public health threat to societies since the complexity of mass vaccination programs, the lack of effective drugs to specifically treat SARS-CoV-2 infected individuals (30–32) and the emergence of VOC predict that virus circulation may last for years (33). A more detailed and broad understanding of the molecular mechanisms underlying the pathogenesis of COVID-19 will have significant implications for developing countermeasures against the virus with particular emphasis to drug discovery, diagnosis and more effective and safe vaccine design (34). The SARS-CoV-2 spike protein is a high glycosylated transmembrane protein assembled as a homotrimer on the virus surface, with three S1 subunits containing the RBD sitting on the top of a trimeric membrane fusion envelope-anchored S2 subunits (35). Residues 319-541 of the spike protein correspond to the RBD, the domain that interacts with the ACE2 receptor of target cells. The RBD undergoes hinge-like conformational movements and constantly switches between an open conformational state (RBD standing-up position) for receptor binding and a closed conformational state (RBD lying-down position). A complex topology corresponds to this dynamism including several co-translational modifications by reason of nine cysteine residues (eight of them forming disulfide bonds) (36), two N-glycosylations at sites N331 and N343 and at least one O-glycosylation at Thr323 and a possible O-glycosylation at Ser325 (19, 35, 37). All these modifications have a relevant role on the spike protein correct folding, dynamics and stability, but glycosylation of RBD seems to be not determinant for its interaction whit ACE2 receptor, since a refolded non-glycosylated rRBD produced in *E. coli* was shown by surface plasmon resonance to bind the receptor (38).

In this study, considering the key role of RBD in viral life cycle, we immunized mice with a rRBD produced in eukaryotic system to establish and characterize novel RBD-specific mouse mAbs. We isolated mAbs that showed extremely potent RBD binding activity and a broad neutralization activity, demonstrated by their ability to block efficiently the infection of SARS-CoV-2 and its major VOC. The performance of these mAbs, including their ability to interact with infectious virus, was probably dependent on the quality of the rRBD used to immunize mice. In fact, we showed that the produced rRBD was correctly folded, N-glycosylated, suitable to interact with ACE2 receptor expressing cells, and recognized by sera of vaccinees and COVID-19 convalescent patients, thus confirming that our HEK293T rRBD reproduced the structural antigenic and functional characteristics of RBD present in native spike protein. We showed that mAbs R590, R64, R71 and R196 do not recognize the *E. coli* rRBD which exposes only linear epitopes as it was produced in denaturing conditions. On the contrary, mAbs react with HEK293T rRBD in ELISA and in WB following SDS-PAGE run under non-reducing conditions. The tridimensional configuration of proteins permits the generation of conformational epitopes formed from discontinuous antigenic determinants and it is largely dependent on disulfide bridges integrity (22). Therefore, reducing agents, such as βME, breaking disulfide bonds can affect the structure of conformational epitopes. Moreover, the mAbs R590, R71 and R196 lose their binding efficiency when HEK293T rRBD is fully N-de-glycosylated. Several plausible mechanisms can be envisaged to speculate how glycosylation could influence antibody binding (39). Glycans could be directly recognized by the antibody or may modify the conformation of the epitope. It is unlikely that our mAbs recognize a glycan antigen, since in this case their reactivity would not be lost altering the conformation of the protein with βME. A possible explanation is that N-glycosylation does not induce significant changes in protein structure, but decreases protein dynamics, leading to an increase in protein stability (40). The consequent conformational equilibrium of the antigen determinant may amplify the binding affinity of the specific antibody (41). Overall, these results suggest that our mAbs recognize conformational, but not linear epitopes on glycosylated RBD. This conclusion is in line with the data reported by Li Y et al. who demonstrated that RBD does not expose linear epitopes (42). Therefore, we did not map the epitopes recognized by our mAbs using a linear peptide library, whereas we attempted to define their binding and functional interactions with RBD. The antibodies R590 and R64 blocked the interaction of HEK293T rRBD with human ACE2 and neutralized infection of VERO E6 using both infectious SARS-CoV-2 and a pseudovirus based on LV expressing Luciferase and pseudotyped with SARS-CoV-2 spike. Instead, R196 and R71 were unable to inhibit the binding of HEK293T rRBD to ACE2 and while R196 was not able to block virus infection in the neutralization assays, R71 showed neutralization activity at high concentration with a mechanism that conceivably does not involve the RBD-ACE2 interaction. In addition, even if all mAbs recognized the native form of the protein expressed on the HEK293T transfected cells, only R590, R71 and R196 but not R64 were able to bind the S1 region of inactivated SARS-CoV-2 supernatants of infected VERO E6 cells in a WB after SDS-PAGE run in non-reducing conditions. Interestingly, R64 immunoprecipitated the spike protein present on lentiviral pseudoviruses, suggesting that the epitope recognized by R64 is susceptible to any structural modification of the spike protein, including those caused by the SDS used for PAGE analysis (21). Based on all these data, we hypothesize that these anti-RBD mAbs bind to different epitopes on RBD: the two different epitopes recognized by R590 and R64 are likely located in the receptor binding site involved in the ACE2 interaction, while the two different epitopes recognized by R71 and R196 are located outside RBM (43).

It is worth noting that mAbs R590, R71 and R196 showed a high binding potency in ELISA and mAbs R590 and R64 also showed a high neutralizing efficiency. This is not surprising, since our mAbs were generated in mice following a prime-boosts schedule with a total of four immunizations at 14 days intervals to favor the affinity maturation of specific antibodies. A similar approach was followed for the isolation of specific human mAbs from vaccinated individuals (44) and differs from approaches based on the isolation of mAbs from convalescent donors, who experienced an antigenic challenge in the limited time-frame of the acute phase of the disease (45–48), that require a larger screening to identify mAbs with high potency.

Of particular interest, and in contrast to many described nAbs (27, 49–51), R590, R64 and R71showed the remarkable capacity to neutralize the major SARS-CoV-2 VOC (Alpha, Beta, Gamma and Delta) in a pseudovirus-based neutralization assay. This result indicates that the rRBD used for immunization exposes epitopes shared by the Wuhan-Hu-1 strains and its VOC. Viral variants must preserve the capacity to infect cells: since the interaction of RBD with the cellular receptor ACE2 is a crucial step in viral entry and infectivity, we may hypothesize that mutations in the RBD sequence, that hamper the generation of the conformational epitopes recognized by mAbs R590 and R64, may also affect the viral fitness reducing the capacity of SARS-CoV-2 to infect cells. Noteworthily, we showed that sera from COVID-19 patients who developed nAbs compete for the binding of our neutralizing mAbs to HEK293T rRBD, suggesting that conformational epitopes of glycosylated RBD are crucial for SARS-CoV-2 infectivity. Since mAbs R590 and R64 also neutralize SARS-CoV-2 VOC, a simple competition ELISA performed using these mAbs would predict whether COVID-19 convalescent or vaccinated individuals developed antibodies able to neutralize SARS-CoV-2 and its VOC. Lastly, the observation that immunization with our HEK293T rRBD induced antibodies neutralizing SARS-CoV-2 and its variants encourages its use for the development of a pan-SARS-CoV-2 subunit vaccine.

This study has some limitations: we did not test the reactivity of our mAbs with SARS-CoV-1, MERS-CoV or other common human coronaviruses and we could not precisely map the conformational epitopes that they recognized using cryo-microscopy, crystal studies, mutagenesis, or other techniques. Also, we did not evaluate the neutralizing activity of mAbs R590 and R64 *in vivo* in animal models. Should animal models confirm the neutralizing activity of these mAbs, the sequence of the immunoglobulin genes of the respective monoclonal hybridoma will easily permit the generation of humanized monoclonal antibodies that would increase the arsenal of “super-antibodies” in the fight against SARS-CoV-2 and its VOC (52).

In conclusion, beside possible implications for the therapy of COVID-19 patients if humanized, the described mAbs can be used for basic research activities to dissect the molecular mechanism of the virus life-cycle by investigating the expression profile and subcellular localization of spike glycoprotein during viral entry, replication, packaging and budding. Moreover, these mAbs could serve as valuable tools for the antigenic diagnosis of COVID-19 or for distinguishing individuals with high titers of nAbs in a simple ELISA format. Therefore, our mAbs may contribute to the advancement in basic and translational research and would accelerate the discovery of drugs targeting virus transmission.

## Data Availability Statement

The raw data supporting the conclusions of this article will be made available by the authors, without undue reservation.

## Ethics Statement

Ethical review and approval was not required for the study on human participants in accordance with the local legislation and institutional requirements. The patients/participants provided their written informed consent to participate in this study. The animal study was reviewed and approved by National Committee for the Protection of Animals Used for Scientific Purposes established by the Animal Care and Welfare Department, Italian Health Ministry.

## Author Contributions

SM, AntC, MC, AI, RT, FT, and PDB: production, purification and characterization of recombinant proteins. SM, RT, AI, and AV: mice immunization, hybridoma production and mAb screening. SM, RT, MC, AntC, and AI: purification and characterization of mAb. MBa, AM, PB, FaM, and ZM: SARS-CoV-2 culture and virus neutralization assay. AndC, MP, AG, PM, and ACar: production of plasmids, lentiviral and retroviral vectors. MBo, AG, MA, and DN: pseudo-virus neutralization assay. CA, SS, MS, and PDB: genetic studies and plasmids design. GV: generation of B16-hACE2 cells. FrM and VE: size exclusion chromatography. RN, SM, AntC, MC, PDB, FaM, MS, ACar, and DN conceived the study and RN, SM, AntC, MC, ACar, and DN wrote the manuscript. All authors contributed to the article and approved the submitted version.

## Funding

This work was funded, and the publication fee was provided by ISS intramural funds, NATO grant #G5817. In addition, this project has received funding from the European Union’s Horizon 2020 research and innovation program under grant agreement no. 681137 (EAVI2020).

## Conflict of Interest

The authors declare that the research was conducted in the absence of any commercial or financial relationships that could be construed as a potential conflict of interest.

## Publisher’s Note

All claims expressed in this article are solely those of the authors and do not necessarily represent those of their affiliated organizations, or those of the publisher, the editors and the reviewers. Any product that may be evaluated in this article, or claim that may be made by its manufacturer, is not guaranteed or endorsed by the publisher.

## Supplementary Material

Supplementary Figure S1 Quantification of rRBD produced in mammalian system. **(A)** Increasing volumes of purified rRBD and known amounts of BSA were resolved on SDS-PAGE. The gel was stained using Krypton fluorescent protein stain. **(B)** Lane profiling of total protein signal showing a cross-section view of each lane rotated 90 degrees. Area of green peaks above the lane is proportional to the amount of protein and its value is reported. Rf, current relative front value. Intensity (Int), average intensity value. * indicate the amount of BSA used to build a standard curve; ** indicate the calculated amount of rRBD based on the BSA standard curve.

Supplementary Figure S2 rRBD production in *E. coli* coli expression system. **(A)** Schematic representation of RBD construct in *E. coli* expression vector. RBD fragment was inserted into BamHI/HindIII restriction sites of pQE30 vector’s multi cloning site (MCS), in frame with the ATG and 6xHis tag. **(B)** Protein purity was analyzed by SDS-PAGE followed by staining with Coomassie blue. Protein identity was confirmed by WB using the anti-RGSHHHH antibody. One of three independent experiments is shown.
