# Supplementary figures and images for: Isolation and Characterization of Mouse Monoclonal Antibodies That Neutralize SARS-CoV-2 and Its Variants of Concern Alpha, Beta, Gamma and Delta by Binding Conformational Epitopes of Glycosylated RBD With High Potency
Source: Front Immunol. 2021 Oct 26;12:750386. doi: 10.3389/fimmu.2021.750386 (PMC8576447; doi:10.3389/fimmu.2021.750386)

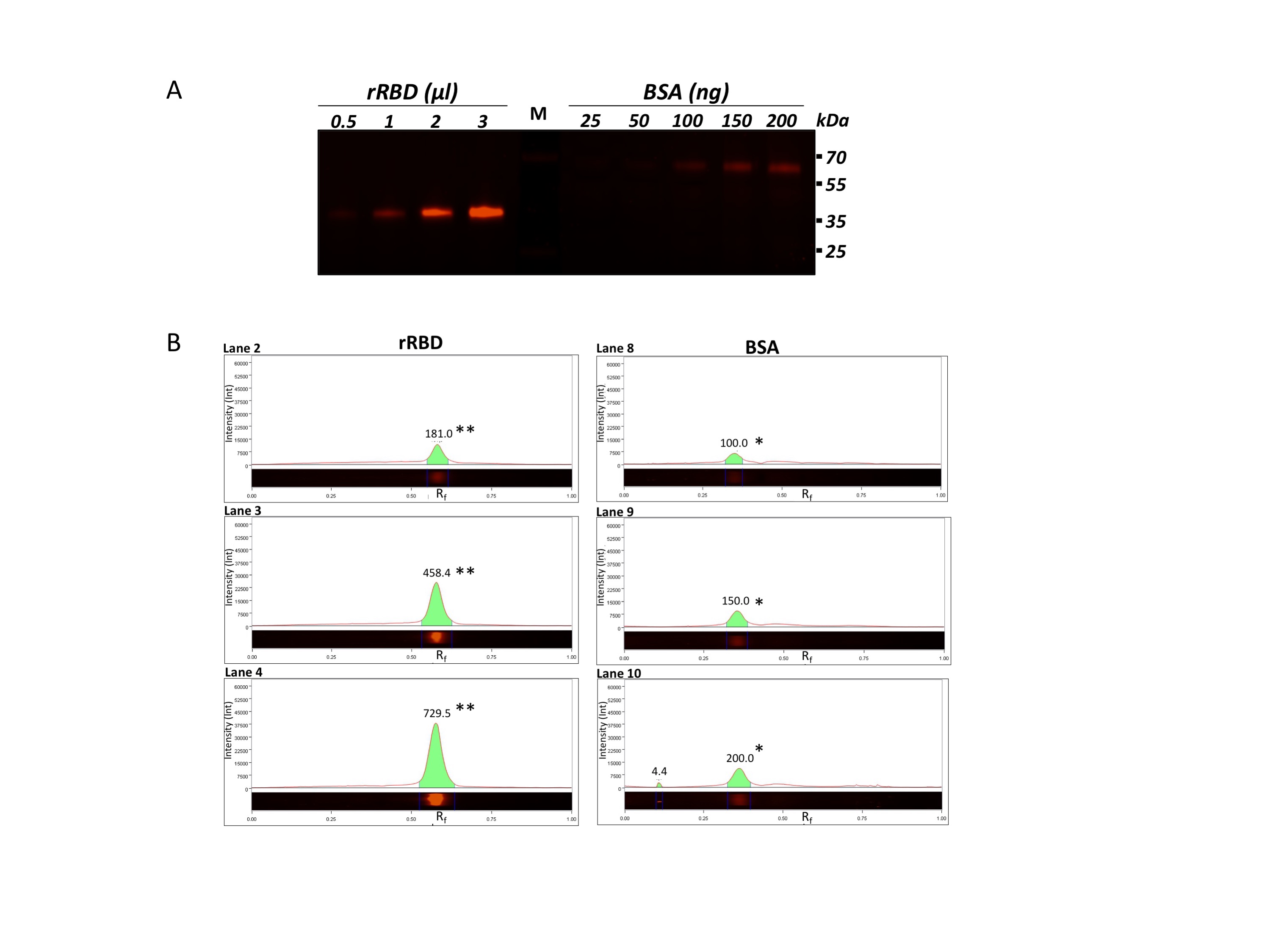

Supplement: Supplementary Figure S1 — Quantification of rRBD produced in mammalian system. (A) Increasing volumes of purified rRBD and known amounts of BSA were resolved on SDS-PAGE. The gel was stained using Krypton fluorescent protein stain. (B) Lane profiling of total protein signal showing a cross-section view of each lane rotated 90 degrees. Area of green peaks above the lane is proportional to the amount of protein and its value is reported. Rf, current relative front value. Intensity (Int), average intensity value. * indicate the amount of BSA used to build a standard curve; ** indicate the calculated amount of rRBD based on the BSA standard curve. [file Image_1.jpeg]

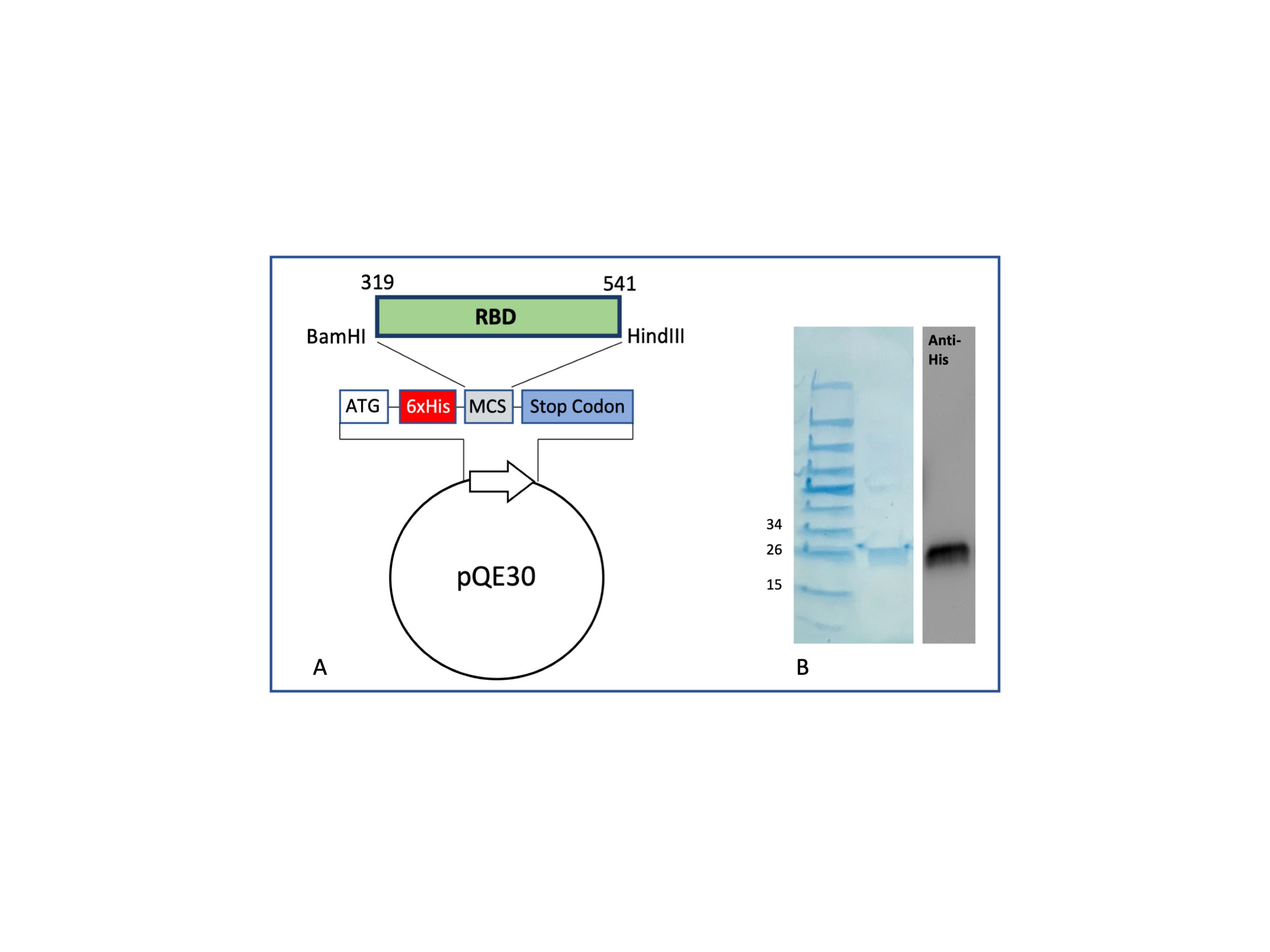

Supplement: Supplementary Figure S2 — rRBD production in E. coli coli expression system. (A) Schematic representation of RBD construct in E. coli expression vector. RBD fragment was inserted into BamHI/HindIII restriction sites of pQE30 vector’s multi cloning site (MCS), in frame with the ATG and 6xHis tag. (B) Protein purity was analyzed by SDS-PAGE followed by staining with Coomassie blue. Protein identity was confirmed by WB using the anti-RGSHHHH antibody. One of three independent experiments is shown. [file Image_2.jpeg]
